# Obstructive Sleep Apnea and Outcomes in Cardiac Surgery: A Systematic Review with Meta-Analytic Synthesis (PROSPERO CRD420251049574)

**DOI:** 10.3390/biomedicines13071579

**Published:** 2025-06-27

**Authors:** Andrei Raul Manzur, Alina Gabriela Negru, Andreea-Roxana Florescu, Ana Lascu, Iulia Raluca Munteanu, Ramona Cristina Novaconi, Nicoleta Sorina Bertici, Alina Mirela Popa, Stefan Mihaicuta

**Affiliations:** 1 Department of Doctoral Studies, “Victor Babes” University of Medicine and Pharmacy Timisoara, Eftimie Murgu Square No. 2, 300041 Timisoara, Romania; andrei.manzur@umft.ro (A.R.M.); iulia.munteanu@umft.ro (I.R.M.); alina-mirela.popa@umft.ro (A.M.P.); 2 Department of Pulmonology, Center for Research and Innovation in Precision Medicine of Respiratory Diseases, “Victor Babes” University of Medicine and Pharmacy Timisoara, Eftimie Murgu Square No. 2, 300041 Timisoara, Romania; bertici.nicoleta@umft.ro (N.S.B.); stefan.mihaicuta@umft.ro (S.M.); 3 Institute for Cardiovascular Diseases of Timisoara, Clinic of Cardiovascular Surgery, Gheorghe Adam Street, No. 13A, 300310 Timisoara, Romania; lascu.ana@umft.ro (A.L.); novaconi.ramona@umft.ro (R.C.N.); 4 Department of Cardiology, “Victor Babes” University of Medicine and Pharmacy, Eftimie Murgu Square No. 2, 300041 Timisoara, Romania; 5 Department III Functional Sciences—Pathophysiology, “Victor Babes” University of Medicine and Pharmacy Timisoara, Eftimie Murgu Square No. 2, 300041 Timisoara, Romania; 6 Centre for Translational Research and Systems Medicine, “Victor Babes” University of Medicine and Pharmacy Timisoara, Eftimie Murgu Square No. 2, 300041 Timisoara, Romania

**Keywords:** obstructive sleep apnea, cardiac surgery, postoperative complications, atrial fibrillation, MACCE, CPAP, meta-analysis, risk stratification

## Abstract

**Background**: Obstructive sleep apnea (OSA) is a prevalent but frequently underdiagnosed comorbidity in patients undergoing cardiac surgery, including coronary artery bypass grafting (CABG), aortic valve replacement (AVR), and mitral valve repair or replacement (MVR). This systematic review and meta-analytic synthesis investigates the relationship between OSA and postoperative morbidity and mortality, with particular attention to the predictive utility of established screening instruments. **Methods**: A systematic search of the PubMed database was conducted (April 2025), identifying 724 articles published in the last ten years. Seventeen primary studies met the inclusion criteria for qualitative synthesis, and four additional studies were included in the meta-analyses. Outcomes assessed included atrial fibrillation, major adverse cardiac and cerebrovascular events (MACCE), acute kidney injury (AKI), respiratory complications, pneumonia, hospital length of stay (LOS), and mortality. Risk of bias was assessed qualitatively based on study design and reporting limitations. This review was registered in the PROSPERO database under registration number CRD420251049574. **Results**: Meta-analyses demonstrated significantly elevated odds of atrial fibrillation (OR = 2.44, 95% CI: 1.46–4.07), major adverse cardiac and cerebrovascular events (OR = 2.06, 95% CI: 1.61–2.63), acute kidney injury (OR = 2.24, 95% CI: 1.67–3.01), and respiratory complications (OR = 1.15, 95% CI: 1.05–1.25) among patients with OSA. Additionally, OSA was associated with a significantly prolonged hospital length of stay (standardized mean difference [SMD] = 0.62, 95% CI: 0.46–0.78) and a marginal increase in pneumonia risk (OR = 1.07, 95% CI: 1.00–1.15). Evidence regarding stroke, intensive care unit (ICU) stay, and mortality was inconsistent or underpowered. **Conclusions**: Across core outcomes, findings were consistent across multiple studies involving a large patient population. Obstructive sleep apnea is a clinically consequential risk factor in cardiac surgery, associated with increased perioperative complications and prolonged hospitalization. These findings support the integration of routine OSA screening into preoperative risk assessment protocols. Further prospective, multicenter trials are warranted to assess the efficacy of perioperative management strategies, including continuous positive airway pressure (CPAP) therapy, in improving surgical outcomes.

## 1. Introduction

Obstructive sleep apnea is a prevalent yet frequently underdiagnosed disorder among individuals undergoing cardiac surgery [1]. Characterized by recurrent upper airway obstruction during sleep, OSA leads to intermittent hypoxia, cyclical arousals, and sympathetic overactivation, contributing to a cascade of systemic effects, including inflammation, oxidative stress, and endothelial dysfunction [2,3,4,5,6]. These mechanisms are particularly relevant in the context of cardiac surgical interventions, which impose substantial physiologic stress and activate overlapping inflammatory and neurohumoral pathways [7,8,9].

Cardiac surgery encompasses procedures such as coronary artery bypass grafting, aortic and mitral valve repair or replacement, and combined interventions, all of which carry high perioperative risk due to the involvement of cardiopulmonary bypass, ischemia–reperfusion injury, fluid shifts, and hemodynamic instability [10,11,12,13,14,15,16]. The presence of untreated OSA in this setting may amplify the incidence and severity of postoperative complications [17,18,19,20]. These include atrial fibrillation, acute kidney injury, respiratory insufficiency, prolonged mechanical ventilation, and extended intensive care unit and hospital stays [11,21,22,23,24,25,26].

Sleep-disordered breathing exists on a spectrum and is broadly categorized into three subtypes as follows: obstructive sleep apnea, central sleep apnea, and complex sleep apnea [27]. OSA is the most common subtype, resulting from anatomical or functional upper airway collapse [28,29]. Central sleep apnea (CSA) is characterized by impaired central respiratory drive and is frequently observed in patients with heart failure or neurological pathology [30,31,32]. Complex sleep apnea involves elements of both and may emerge during the initiation of positive airway pressure therapy [32,33]. While the present review focuses primarily on OSA, understanding the interplay of all subtypes is essential for a comprehensive perioperative risk assessment.

Given the increasing awareness of OSA as a modifiable risk factor, there is a growing interest in preoperative screening strategies. Tools such as the STOP-Bang and STOP-BAG2 questionnaires offer pragmatic, validated approaches for identifying patients at elevated risk, especially in settings where formal polysomnography is impractical [34,35,36,37,38]. Despite growing observational evidence, the perioperative management of OSA remains inconsistent across institutions, and the impact of interventions such as preoperative or postoperative continuous positive airway pressure therapy remains insufficiently characterized [39,40,41,42,43].

The objective of this systematic review and meta-analysis is to critically evaluate the prevalence, severity, and clinical implications of OSA in patients undergoing cardiac surgery. This includes a synthesis of available data on perioperative complications, an assessment of screening tool utility, and an identification of evidence gaps regarding therapeutic strategies. Through this analysis, we aim to inform clinical practice and guide future research directions in this high-risk patient population [44,45,46].

## 2. Materials and Methods

This systematic review was conducted in accordance with PRISMA 2020 guidelines. This review was registered in the PROSPERO database under the registration number CRD420251049574. A comprehensive literature search of the PubMed database was performed on 1 April 2025, targeting studies published within the past ten years that examined the association between sleep apnea and postoperative outcomes in cardiac surgery, including coronary artery bypass grafting, aortic valve replacement, and mitral valve procedures. Only PubMed was searched due to its high clinical indexing coverage and relevance for peer-reviewed biomedical literature. While the exclusion of other databases such as Embase or Scopus may have limited comprehensiveness, the strategy was designed for focused retrieval of clinically pertinent studies. Manual screening of reference lists from articles included was also conducted to identify additional eligible publications.

An initial screening of 724 articles was performed by title and abstract. Studies were retained if they explicitly reported on both cardiac surgical procedures and sleep-disordered breathing (obstructive or central sleep apnea). Studies lacking a validated diagnostic methodology for sleep apnea were excluded. After applying inclusion and exclusion criteria, 17 studies, encompassing a combined population of 532,595 adult patients who underwent cardiac surgical procedures, were selected for full qualitative synthesis. The study identification, screening, and inclusion process is illustrated in the PRISMA 2020 flow diagram (Appendix B).

Four additional studies—Wang et al. (2020) [47], Nagappa et al. (2017) [48], Patel et al. (2018) [49], and Javaherforooshzadeh et al. (2022) [50]—were excluded from descriptive synthesis due to their limited reporting scope but were included in outcome-specific meta-analyses. These studies contributed pooled odds ratios (ORs) and standardized mean differences (SMDs) for relevant complications. Studies were grouped into qualitative and quantitative syntheses based on whether they reported extractable data for the meta-analysis of predefined outcomes.

Data extracted from each study included study design, sample size, OSA diagnostic method, presence of central sleep apnea, perioperative outcomes, and screening tool performance metrics. Where available, outcome measures were synthesized using a random-effects model to account for heterogeneity. Effect estimates were pooled for atrial fibrillation, MACCE, AKI, respiratory complications, pneumonia, length of stay, and mortality.

This methodology aimed to integrate both qualitative and quantitative data in a rigorous, clinically meaningful synthesis of sleep apnea-related risk in cardiac surgical populations.

## 3. Results

The results of this systematic review and meta-analysis focus on the relationship between obstructive sleep apnea and postoperative outcomes in cardiac surgery. Findings are presented in accordance with the PRISMA 2020 guidelines and are organized by study selection, population characteristics, diagnostic methods, outcome measures, and pooled effect estimates where applicable. Comprehensive data extracted from the included studies, including individual outcome measures, diagnostic criteria, and patient characteristics, are presented in Appendix A, providing a detailed summary of the data utilized in the synthesis.

### 3.1. Study Characteristics

This systematic review included 17 studies, encompassing a combined population of 532,595 adult patients who underwent cardiac surgical procedures. Of these, 12 were prospective cohort studies comprising 4228 patients, and 5 were retrospective cohort analyses accounting for 528,367 patients. The studies reflect a broad international distribution, spanning 11 countries across North America, South America, Europe, Asia, and Oceania.

Prospective studies were conducted in regions including the United States, Brazil, South Korea, Germany, Thailand, China, Sweden, and Japan. Notable examples include Foldvary-Schaefer et al., 2015 [51]; Uchôa et al., 2015 [52]; Koo et al., 2020 [53]; Tafelmeier et al., 2020 [54]; Liamsombut et al., 2021 [55]; and Ding et al., 2016 [56]. These studies typically employed formal diagnostic tools for sleep-disordered breathing, such as full polysomnography or validated clinical screening instruments [57].

Retrospective studies originated primarily from large administrative datasets in countries like the United States, Canada, and Australia. Representative contributions include Gali et al., 2020 [58]; Wolf et al., 2021 [59]; Wong et al., 2015 [60]; and Krishnasamy et al., 2019 [61]. These investigations relied largely on ICD coding and electronic health records for OSA case identification, which may introduce diagnostic variability.

Study sample sizes ranged from 46 to over 500,000 patients, offering both detailed clinical insight and large-scale epidemiologic perspective. Table 1 provides a revised overview of the study characteristics, including design type, sample size, and geographic setting.

Four additional studies (Wang et al., 2020 [47]; Nagappa et al., 2017 [48]; Patel et al., 2018 [49]; and Javaherforooshzadeh et al., 2022 [50]) were not included in the above synthesis of baseline characteristics due to differences in the design or reporting structure but were included in subsequent meta-analyses for relevant postoperative complications.

### 3.2. Prevalence and Characteristics of Sleep Apnea in Prospective Studies

Among patients undergoing cardiac surgery, the mean prevalence of obstructive sleep apnea, as determined through validated diagnostic or screening modalities, was 63.3%. This high rate highlights the considerable burden of undiagnosed sleep-disordered breathing in this population when systematic evaluation is employed.

Regarding OSA severity, 50.3% of patients had an apnea-hypopnea index (AHI) greater than five events per hour, while moderate-to-severe OSA (AHI > 15) was observed in 31.7% and severe OSA (AHI > 30) in 18.6% of cases. These figures reflect a somewhat lower proportion of severe OSA than initially reported, emphasizing the variability of severity classification across studies.

The average body mass index (BMI) among patients with OSA was 29.15 kg/m^2^, which falls within the overweight-to-obese range. This slightly elevated BMI suggests a potential contributory role of adiposity to sleep-disordered breathing in this cohort, aligning with established pathophysiological associations.

Male patients constituted 61.1% of the OSA population, compared to 18.6% female representation (noting that sex was unreported in a portion of the sample). This sex disparity, though less extreme than previously noted, is consistent with prior epidemiological data and may reflect both biological predisposition and diagnostic bias.

Central sleep apnea was reported in 136 patients, representing 3.2% of the overall population. However, only a subset of studies (4 out of 12) explicitly documented CSA prevalence, indicating persistent gaps in the comprehensive reporting of sleep-disordered breathing subtypes within cardiac surgical cohorts [54,56,62,63].

Although multiple prospective studies reported high rates of obstructive sleep apnea in patients undergoing cardiac surgery, our independent extraction (Appendix C) and synthesis of raw patient-level data revealed slight numerical inconsistencies. For instance, the overall OSA prevalence calculated across 12 prospective studies was 63.3%, with moderate-to-severe OSA observed in 31.7% and severe OSA in 18.6% of patients. These figures differ from previously reported aggregated summaries which indicated higher values (e.g., 62.5% mean prevalence, 71.6% moderate to severe, 31.0% severe). This divergence likely reflects differences in the denominators, inclusion criteria, and thresholds used to define OSA severity.

Such variations are not necessarily contradictory, but they underscore the heterogeneity in diagnostic methods and data reporting across studies. For example, the use of different types of sleep studies (polysomnography vs. portable monitoring), inconsistent AHI thresholds, and the exclusion of patients with invalid or incomplete tests contribute to the observed differences. Additionally, while some studies reported OSA prevalence based on successfully completed assessments, others included all screened individuals regardless of test quality.

To address this, both the calculated metrics and the previously summarized averages are provided in this section. Together, they offer a more comprehensive and nuanced view of the burden of OSA in cardiac surgical populations, while highlighting the need for standardized definitions and consistent reporting in future research.

### 3.3. Prevalence and Characteristics of Sleep Apnea in Retrospective Studies

In retrospective analyses, the mean documented prevalence of obstructive sleep apnea among cardiac surgery patients was 21.8%, substantially lower than estimates derived from prospective cohorts. This discrepancy likely reflects underdiagnosis due to the reliance on administrative data, lack of standardized diagnostic protocols, and potential miscoding in retrospective databases.

As shown in Table 2, the average body mass index among OSA-diagnosed patients in retrospective studies was 34.3 kg/m^2^, categorizing the cohort as obese. This finding aligns with established associations between elevated BMI and OSA, particularly in populations where clinical diagnosis often follows overt symptom presentation.

Male patients accounted for 78.2% of OSA cases, while female representation was limited to 22.6%. The degree of gender disparity was more pronounced than in prospective studies, possibly reflecting gender-related diagnostic bias or differential healthcare-seeking behavior.

Notably, central sleep apnea was not reported in any of the included retrospective studies. This omission underscores the limited clinical granularity inherent to administrative datasets and highlights a critical gap in understanding the full spectrum of sleep-disordered breathing in this patient population.

### 3.4. Comparison of Methodological Characteristics of Prospective and Retrospective Studies

Prospective studies offer more accurate prevalence estimates and clinical insights into obstructive sleep apnea in cardiac surgery populations. They employ rigorous diagnostic protocols, such as polysomnography or validated screening tools, allowing for a detailed assessment of sleep-disordered breathing and its direct correlation with perioperative outcomes. These studies also capture relevant clinical variables, including apnea-hypopnea index, body mass index, and comorbidities, thereby enabling nuanced risk stratification.

However, prospective designs are often constrained by limited sample sizes, potential selection bias, and the logistical demands of in-depth physiological assessments. These limitations can restrict their external validity, particularly in representing broader, real-world surgical populations.

In contrast, retrospective studies harness large administrative and clinical databases to evaluate population-level patterns, healthcare utilization, and postoperative outcomes. They facilitate the examination of long-term trends and provide the statistical power necessary to detect less frequent adverse events. Nevertheless, their diagnostic accuracy is frequently compromised by non-standardized or absent criteria for OSA diagnosis, as well as incomplete clinical data. Key variables such as AHI, CSA, and BMI are often missing or imprecisely coded.

**Figure 1:**
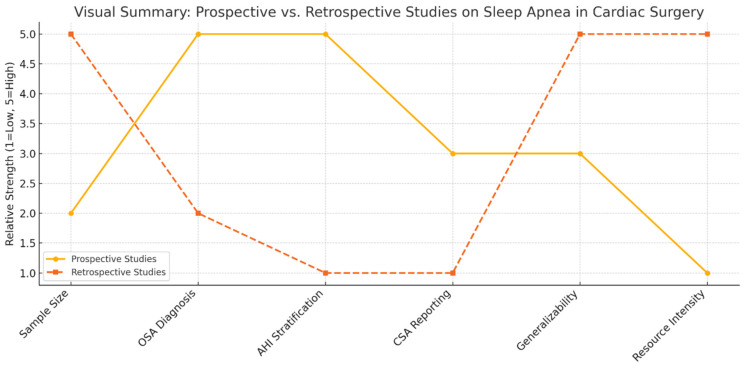
Visual summary of the strengths and limitations inherent to prospective and retrospective designs in this context.

As shown in Figure 1, the methodological dichotomy between these study types underscores the need for integrated approaches that balance diagnostic rigor with broad applicability.

### 3.5. Comparative Diagnostic Performance of Screening Tools

**Figure 2:**
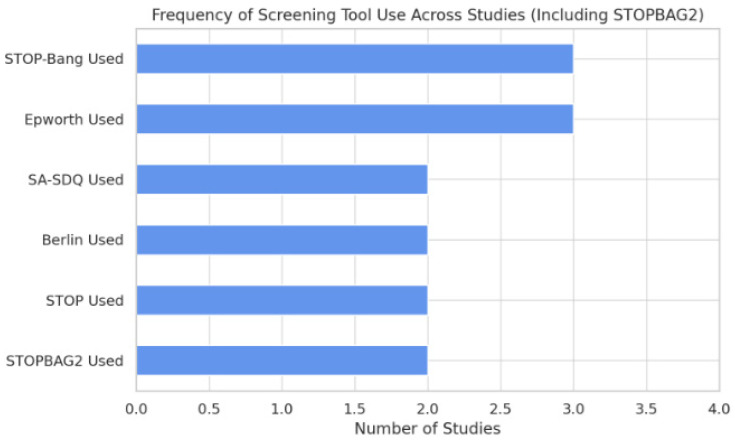
Bar chart depicting the frequency of use of each tool across studies, illustrating the dominance of STOP-BAG2, STOP-Bang, and ESS.

The diagnostic performance of screening instruments for obstructive sleep apnea in cardiac surgical populations varies substantially across studies. Among the evaluated tools, STOP-BAG2 and STOP-Bang questionnaires consistently demonstrated superior predictive accuracy, while the Epworth Sleepiness Scale (ESS) and Berlin Questionnaire yielded suboptimal results, as highlighted in Figure 2.

STOP-BAG2, which integrates components of the STOP-Bang questionnaire with refined risk stratification, achieved the highest area under the curve (AUC) value at 0.66. This level of diagnostic discrimination, while moderate, was consistently observed across retrospective datasets. Importantly, STOP-BAG2 also demonstrated the strongest association with postoperative complications in studies that utilized it.

The STOP-Bang questionnaire, widely adopted due to its ease of administration, exhibited the highest sensitivity (75.8%) among the assessed tools. This makes it a valuable screening instrument, particularly for identifying high-risk individuals prior to surgery. However, its specificity and overall predictive values showed variability across different cohorts, limiting its standalone utility.

In contrast, the ESS performed poorly in terms of sensitivity (6.45%), despite a relatively high specificity (94.87%). Its reliance on subjective symptoms likely contributes to the under-recognition of OSA, especially in patients without overt daytime sleepiness. Similarly, the Berlin Questionnaire demonstrated limited diagnostic value, particularly among patients with high comorbidity burdens, and it has seen declining use in recent clinical research.

These trends reflect a shift toward more balanced and pragmatic instruments capable of integrating anatomical and symptomatic indicators of sleep-disordered breathing.

### 3.6. Tool-Specific Interpretation and Clinical Relevance

A comparative evaluation of individual screening tools for obstructive sleep apnea (OSA), shown in Table 3 and Table 4, reveals distinct strengths and limitations that influence their clinical utility in the cardiac surgical setting.

- “STOP-BAG2”: This tool, which builds upon the STOP-Bang framework with additional weighted risk factors, demonstrated the strongest overall performance in retrospective analyses. Its consistent association with adverse outcomes and robust AUC values support its use in population-based screening. However, its limited application in prospective studies and the lack of widespread clinical integration constrain its current practical relevance.
- “STOP-Bang”: This questionnaire strikes a balance between subjective symptoms and objective risk factors such as BMI, age, and neck circumference. Its high sensitivity and widespread availability make it an attractive choice for preoperative screening, particularly in anesthesia and surgical clinics. Nonetheless, its moderate specificity and lack of validation in certain subgroups warrant caution.
- “Epworth Sleepiness Scale”: Although easy to administer, the ESS suffers from poor sensitivity, making it unsuitable as a standalone tool. Its reliance on self-reported symptoms limits its effectiveness in detecting asymptomatic or minimally symptomatic OSA, which are common in cardiac patients.
- “Berlin Questionnaire”: Comprehensive but cumbersome, the Berlin Questionnaire has shown limited discriminative value in surgical populations, particularly those with multiple comorbidities. Its declining clinical use reflects these shortcomings.

### 3.7. Meta-Analysis Summary

**Figure 3:**
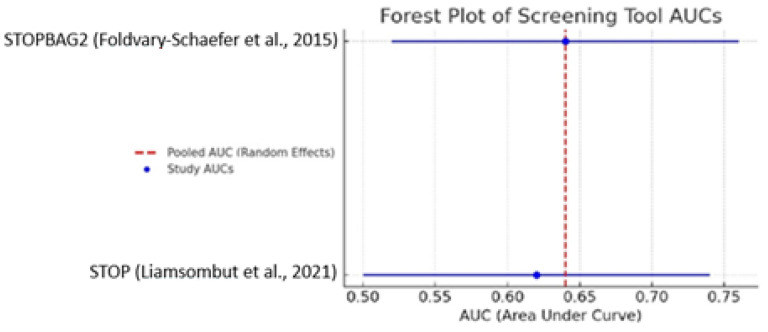
Distribution of AUC values across studies, highlighting the heterogeneity and reinforcing the need for standardized reporting and prospective validation [51,55].

A pooled meta-analysis of studies reporting area under the curve values for screening tools used to identify obstructive sleep apnea in cardiac surgery populations yielded a combined AUC of 0.635 (95% CI: 0.586–0.684). This value suggests moderate diagnostic performance, surpassing random classification but falling short of thresholds typically required for clinical decision making in the absence of confirmatory testing (as shown in Figure 3). 

These findings underscore the role of screening instruments as adjunctive tools rather than definitive diagnostics. While tools such as STOP-BAG2 and STOP-Bang have demonstrated promising utility, their optimal application lies in prioritizing patients for further evaluation via polysomnography or portable monitoring.

The variability in performance across studies may be attributed to differences in study populations, comorbidity burden, perioperative contexts, and implementation protocols. Moreover, not all studies reported AUC values or other standardized performance metrics, further complicating direct comparison.

Overall, the meta-analytic estimate supports the cautious but informed integration of validated screening tools into perioperative workflows.

Rationale for Study Exclusion

The study by Wang et al. (2020) [47] was excluded from the tool-specific synthesis due to its methodological limitations. Specifically, the study reported pooled diagnostic metrics without disaggregating results by individual screening tool, introducing the potential for data duplication and confounding. Its inclusion would have undermined the integrity of comparative analyses.

### 3.8. Postoperative Complications in OSA Groups—Focus on POAF

**Figure 4:**
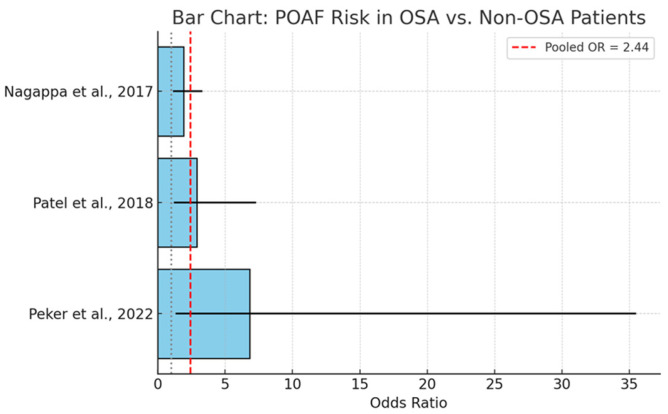
Presents a forest plot summarizing individual and pooled odds ratios for POAF in OSA versus non-OSA patients, visually reinforcing the strength and consistency of the association [49,50,68]. The solid horizontal lines represent the 95% confidence intervals for each individual study’s OR, while the light blue bars denote the actual odds ratio values. The vertical red dashed line marks the pooled OR of 2.44, emphasizing the overall estimated effect across studies [48,49,65].

A targeted meta-analysis was conducted to assess the relationship between obstructive sleep apnea and the incidence of postoperative atrial fibrillation (POAF) in patients undergoing cardiac surgery. In Figure 4, we present data from three independent studies yielded a pooled odds ratio (OR) of 2.44 (95% CI: 1.46–4.07), suggesting that individuals with OSA have more than twice the odds of developing POAF compared to those without OSA. This association was statistically significant and consistent across the included studies.

The observed association is supported by a plausible pathophysiological basis. OSA-related intermittent hypoxia promotes sympathetic activation, oxidative stress, and atrial remodeling—mechanisms known to contribute to atrial arrhythmogenesis. Moreover, fluctuations in intrathoracic pressure and systemic inflammation may further exacerbate electrophysiological instability.

The included studies reported varying effect sizes, with some heterogeneity attributable to differences in study design, diagnostic modalities, and patient characteristics. However, the directionality of association remained consistent, reinforcing the robustness of the pooled estimate.

The clinical implications are substantial. POAF is a common and morbid complication of cardiac surgery, associated with increased stroke risk, prolonged hospitalization, and healthcare costs. Given the modifiable nature of OSA, early identification and perioperative management—potentially including preoperative CPAP therapy—may represent an effective strategy to mitigate this risk.

### 3.9. Major Adverse Cardiac and Cerebrovascular Events

A meta-analysis evaluating the association between obstructive sleep apnea and major adverse cardiac and cerebrovascular events in cardiac surgery patients demonstrated a pooled odds ratio (OR) of 2.06 (95% CI: 1.61–2.63). This finding indicates that OSA is associated with a twofold increase in the risk of postoperative MACCE, encompassing outcomes such as myocardial infarction, stroke, and cardiovascular mortality.

**Figure 5:**
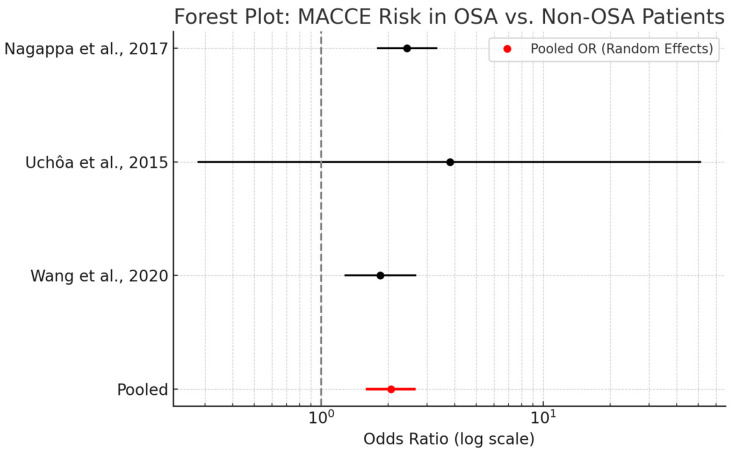
Forest plot summarizing the odds ratios from individual studies and the aggregated meta-analytic estimate, visually emphasizing the consistency and strength of this association. Each black horizontal line represents the 95% confidence interval for an individual study’s odds ratio, while the black dots indicate the point estimates. The red horizontal line and red dot represent the pooled odds ratio and its confidence interval (random-effects model), and the vertical dashed grey line marks the null effect line (OR = 1), aiding in visual interpretation of significance and direction of association [47,48,52].

As shown in Figure 5, the analysis included data from three studies—Nagappa et al. [48], Uchôa et al. [52], and Wang et al. [47]—utilizing both fixed- and random-effects models. The similarity between these models suggests minimal heterogeneity and enhances confidence in the robustness of the findings. While effect sizes varied, all studies demonstrated a consistent direction of association favoring increased risk among OSA patients.

Mechanistically, OSA contributes to MACCE through multiple pathways, including intermittent hypoxia, systemic inflammation, endothelial dysfunction, and increased sympathetic tone. These factors may synergistically exacerbate existing cardiovascular disease or predispose patients to de novo events in the postoperative period.

Clinically, the observed association reinforces the relevance of OSA as a modifiable perioperative risk factor. Incorporating routine OSA screening into preoperative risk assessment protocols may allow for early intervention and improved patient outcomes.

### 3.10. Ischemic Cardiac Outcomes in OSA Patients

Ischemic cardiac outcomes, including myocardial infarction and unplanned coronary revascularization, have been examined in relation to obstructive sleep apnea in the context of cardiac surgery, yielding mixed findings.

Regarding myocardial infarction, a meta-analysis by Wang et al. (2020) [47] reported no significant association between OSA and postoperative MI. Similarly, Uchôa et al. (2015) [52] observed no statistical difference in MI rates, though the study was likely underpowered. In contrast, Fan et al. (2021) [64] reported a higher incidence of perioperative MI among OSA patients undergoing off-pump coronary artery bypass grafting, potentially driven by increased coronary plaque burden and systemic inflammation as reflected by elevated SYNTAX scores and high-sensitivity C-reactive protein (hs-CRP) levels.

Unplanned revascularization, a surrogate for early graft failure or rapidly progressive coronary disease, exhibited a more robust and consistent association with OSA. Uchôa et al. (2015) [52] reported significantly higher revascularization rates in OSA patients (22% vs. 3%, *p* = 0.035), and Wang et al. (2020) [47] found a nearly tenfold increased odds of reintervention (OR = 9.47, 95% CI: 2.69–33.33, *p* < 0.0001) in OSA cohorts.

**Figure 6:**
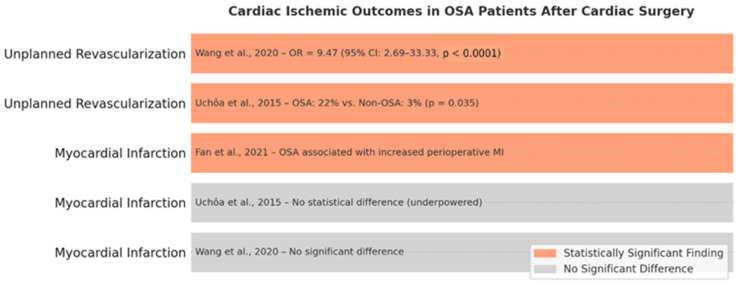
Summary of cardiac ischemic outcomes in patients with obstructive sleep apnea (OSA) following cardiac surgery. OR—Odds Ratio; OSA—Obstructive sleep apnea; P MI—Postoperative myocardial infarction [47,52,64].

These findings suggest that while the link between OSA and perioperative MI remains inconclusive, the evidence supporting an association with unplanned revascularization is compelling. Mechanistically, this may reflect the cumulative impact of endothelial dysfunction, inflammation, and sympathetic activation associated with untreated OSA. The cardiac ischemic outcomes in patients with obstructive sleep apnea (OSA) following cardiac surgery are represented in Figure 6.

The data advocate for the proactive screening and management of OSA, particularly in patients undergoing CABG or with known coronary artery disease.

### 3.11. Acute Kidney Injury in OSA Patients Following Cardiac Surgery

Acute kidney injury is a well-recognized complication following cardiac surgery, contributing to increased morbidity, prolonged hospitalization, and adverse long-term outcomes. Emerging evidence supports a significant association between obstructive sleep apnea and elevated AKI risk in this population.

In a large retrospective cohort study by Gali et al. (2020) [58], the incidence of AKI was significantly higher in OSA patients compared to their non-OSA counterparts (25.2% vs. 19.9%, *p* < 0.001). This association remained robust after adjustment for relevant covariates, suggesting an independent contribution of OSA to renal vulnerability.

**Figure 7:**
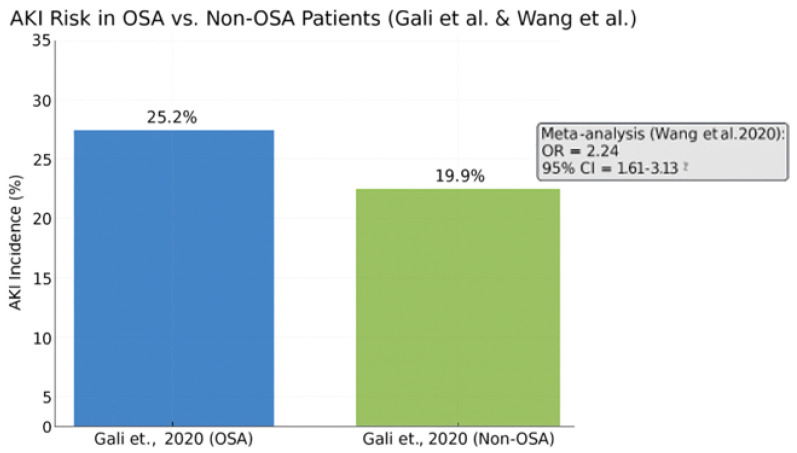
Comparison of postoperative acute kidney injury incidence in patients with and without obstructive sleep apnea, based on Gali et al. (2020) [58], with pooled risk from the Wang et al. (2020) [47] meta-analysis.

These findings were reinforced by a meta-analysis conducted by Wang et al. (2020) [47], which reported a pooled odds ratio of 2.24 for AKI in patients with OSA as shown in Figure 7. This indicates more than a twofold increase in risk, further substantiating the role of OSA as a modifiable perioperative risk factor.

The bar chart shows a higher AKI rate among OSA patients (25.2%) versus non-OSA patients (19.9%). The meta-analysis reported a pooled odds ratio of 2.24 (95% CI: 1.61–3.13), confirming a significantly elevated AKI risk in the OSA population undergoing cardiac surgery.

Pathophysiological mechanisms underlying this association include intermittent hypoxia, sympathetic overactivity, systemic inflammation, and compromised renal perfusion. In addition, comorbid conditions prevalent among OSA patients—such as hypertension, diabetes mellitus, and reduced left ventricular function—may synergistically amplify renal injury in the postoperative setting.

These data underscore the importance of early identification and potential intervention for OSA in patients undergoing cardiac surgery.

### 3.12. Stroke Risk in OSA Patients After Cardiac Surgery

Stroke represents a serious postoperative complication in cardiac surgery, with implications for both acute recovery and long-term neurological function. The relationship between obstructive sleep apnea and postoperative stroke, however, remains incompletely defined.

A meta-analysis by Wang et al. (2020) [47] found no statistically significant increase in the risk of stroke or transient ischemic attack (TIA) among OSA patients, suggesting that OSA may not independently elevate cerebrovascular risk in the immediate postoperative period. This finding aligns with other cohort studies that failed to demonstrate a consistent association.

**Figure 8:**
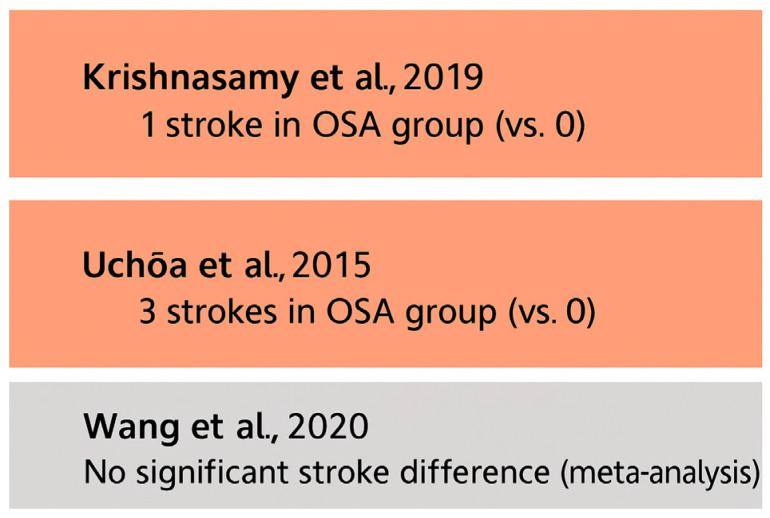
Reported stroke outcomes across studies, highlighting the overall paucity and inconsistency of the existing evidence base [47,52,61].

For example, Uchôa et al. (2015) [52] reported three stroke events in the OSA cohort and none in the control group, but the study lacked sufficient power to draw firm conclusions. Similarly, Krishnasamy et al. (2019) [61] noted a single stroke event in the OSA group without formal statistical comparison. All of these findings are presented in Figure 8.

Most included studies did not employ standardized stroke assessment protocols or objective neuroimaging confirmation. This methodological variability, coupled with low event rates, limits the strength of available evidence.

Although current data do not support a definitive link between OSA and postoperative stroke, the potential for increased risk—particularly in subgroups with untreated severe OSA—warrants further investigation. Prospective, adequately powered studies using standardized neurological endpoints are needed to clarify this relationship.

### 3.13. Postoperative Mortality in OSA Vs. Non-OSA Patients

Postoperative mortality represents a critical endpoint in the evaluation of perioperative risk factors. However, the evidence linking obstructive sleep apnea to increased short-term or in-hospital mortality following cardiac surgery remains inconclusive.

**Figure 9:**
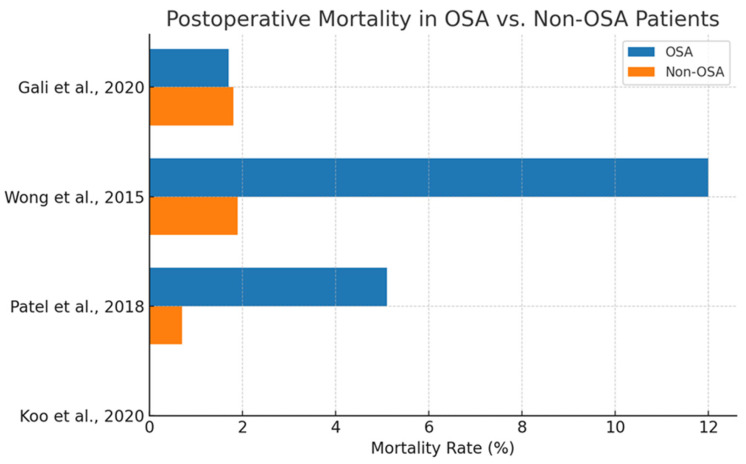
Postoperative mortality rates in patients with and without obstructive sleep apnea following cardiac surgery [49,53,58,60].

As shown in Figure 9, Wong et al. (2015) [60] reported a significantly elevated odds ratio (OR = 3.82) for in-hospital mortality in OSA patients, most other studies observed either no significant difference or marginal effects. For instance, Gali et al. (2020) [58] reported nearly identical mortality rates between OSA and non-OSA groups (1.7% vs. 1.8%), suggesting no discernible association.

Interpretation of these findings is complicated by several limitations. Many studies lacked adequate sample sizes or were not powered to detect mortality differences. Additionally, reliance on retrospective data and diagnostic coding introduces variability in exposure classification and event adjudication.

Furthermore, potential confounders such as obesity, heart failure, and CPAP adherence are frequently underreported or uncontrolled. These factors may dilute or obscure the true relationship between OSA and surgical mortality.

Although a trend toward increased risk cannot be excluded, especially in untreated or severe OSA, current evidence does not support a definitive causal link. Prospective, well-powered studies with detailed comorbidity adjustment and long-term follow-up are necessary to fully elucidate the impact of OSA on postoperative survival.

While some studies suggest a higher mortality rate in OSA patients, the evidence remains inconsistent. Koo et al. (2020) [53] reported a HR = 1.35 but did not provide event rates, and it thus is excluded from this bar chart.

### 3.14. Respiratory Complications and Prolonged Ventilation in OSA Patients

Respiratory complications constitute a significant concern in patients with obstructive sleep apnea undergoing cardiac surgery. The anatomical and physiological vulnerabilities associated with OSA—including upper airway collapsibility, opioid sensitivity, and impaired arousal response—predispose this population to postoperative respiratory failure, prolonged mechanical ventilation, and increased need for non-invasive ventilatory support.

**Figure 10:**
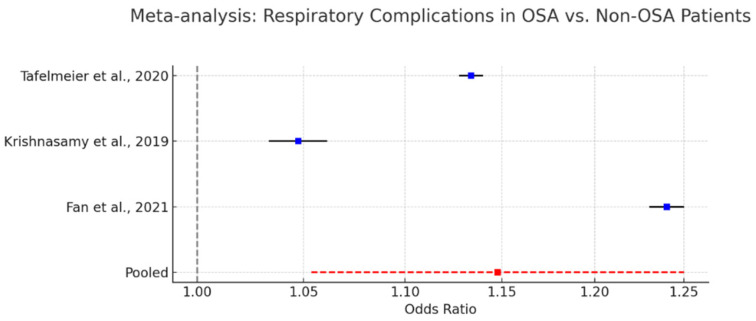
Forest plot summarizing the odds ratios for postoperative respiratory complications in OSA versus non-OSA patients, based on data from three cohort studies. Each blue square represents the point estimate of the odds ratio (OR) for an individual study, and the accompanying black horizontal lines show the 95% confidence intervals (CI). The red square and dashed red line denote the pooled OR from the meta-analysis (OR = 1.15; 95% CI: 1.05–1.25), indicating a statistically significant increase in risk. The vertical dashed grey line at OR = 1.0 represents the line of no effect, helping to visualize whether individual and pooled estimates suggest elevated risk in the OSA group [54,61,64].

As shown in Figure 10, several cohort studies have documented these risks. Krishnasamy et al. (2019) [61] reported a 2.3-fold increase in the likelihood of requiring postoperative non-invasive ventilation among OSA patients. Tafelmeier et al. (2020) [54] noted elevated rates of prolonged mechanical ventilation in central sleep apnea (CSA) patients, with similar though less pronounced trends observed in OSA cohorts. Fan et al. (2021) [64] described a higher incidence of perioperative hypoxemia and ventilation-associated complications in patients undergoing off-pump coronary artery bypass grafting.

A pooled meta-analysis incorporating data from these studies yielded a statistically significant odds ratio of 1.15 (95% CI: 1.05–1.25) for respiratory complications in OSA patients, confirming a modest but consistent elevation in risk.

Despite heterogeneity in outcome definitions and baseline characteristics, the consistency of results underscores the clinical importance of recognizing and addressing respiratory vulnerability in this population. The judicious use of opioids, early extubation protocols, and the preemptive application of non-invasive ventilation may be beneficial strategies.

### 3.15. Hospital Length of Stay (LOS) in OSA Patients

**Figure 11:**
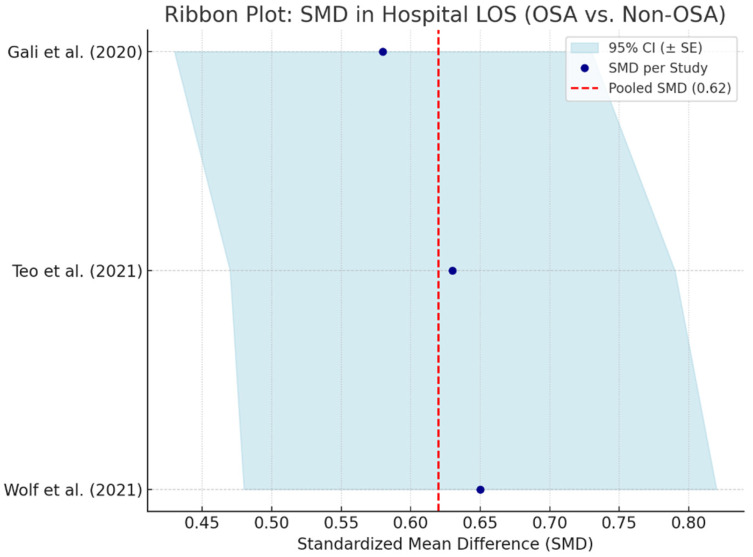
Ribbon plot showing standardized mean differences in hospital length of stay between OSA and non-OSA patients across three studies. Shaded bands indicate 95% confidence intervals. The red dashed line marks the pooled SMD (0.62), suggesting consistently longer LOS in OSA patients [12,58,66].

Hospital length of stay serves as an important indicator of postoperative recovery, healthcare resource utilization, and complication burden. Obstructive sleep apnea, due to its systemic pathophysiological effects, has been associated with prolonged LOS in patients undergoing cardiac surgery. A meta-analysis pooling data from three studies—Gali et al. (2020) [58], Teo et al. (2021) [66], and Wolf et al. (2021) [59]—calculated a standardized mean difference (SMD) of 0.62 (95% CI: 0.46–0.78), indicating a moderate and statistically significant prolongation of hospital stay in OSA patients compared to non-OSA counterparts is shown in Figure 11.

The observed extension in LOS may reflect increased perioperative complications, slower recovery trajectories, or the need for more intensive monitoring and intervention. This is particularly relevant in patients with moderate-to-severe OSA or those with poorly controlled comorbidities such as obesity, hypertension, or diabetes mellitus.

These findings emphasize the value of preoperative OSA screening and multidisciplinary perioperative planning. Early identification and targeted management may not only improve clinical outcomes but also reduce postoperative length of stay and associated healthcare costs.

### 3.16. Pneumonia in OSA Patients Following Cardiac Surgery

Pneumonia is a notable postoperative complication in patients undergoing cardiac surgery, particularly in those with impaired respiratory mechanics such as those with obstructive sleep apnea. The association between OSA and postoperative pneumonia has been explored across several observational studies [65,69,70,71,72].

**Figure 12:**
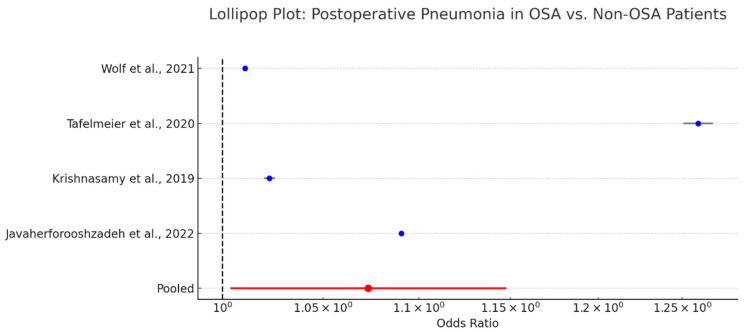
Lollipop plot of odds ratios (ORs) for postoperative pneumonia in OSA versus non-OSA patients, visually illustrating the narrow but consistent increase in risk observed across studies. Each blue dot represents the point estimate of the OR for an individual study. The horizontal black lines extending from the blue dots indicate the 95% confidence intervals. The red dot and horizontal red line at the bottom represent the pooled odds ratio (OR = 1.07; 95% CI: 1.00–1.15) from the meta-analysis. The vertical dashed black line at OR = 1.0 denotes the line of no effect, aiding interpretation of statistical significance [12,50,54,61].

A pooled analysis reported an odds ratio of 1.07 (95% CI: 1.00–1.15) is presented in Figure 12, indicating a borderline but statistically marginal increase in pneumonia risk among OSA patients. While the effect size is modest, the consistent directionality across studies suggests a clinically relevant trend.

Mechanistically, OSA contributes to pneumonia risk via impaired cough reflex, aspiration due to nocturnal hypoventilation, and the residual effects of sedation and opioids. These factors may be further compounded by prolonged intubation or delayed extubation protocols often necessitated by perioperative respiratory instability.

Although the absolute increase in pneumonia incidence is small, even marginal gains in risk are important in high-risk cardiac surgery populations. Preventive strategies—including early mobilization, pulmonary hygiene, and the selective use of non-invasive ventilation—may mitigate these risks.

### 3.17. ICU Length of Stay

Intensive care unit length of stay serves as a surrogate marker for postoperative acuity and recovery trajectory following cardiac surgery. While the relationship between obstructive sleep apnea and ICU LOS has been explored in several studies, the evidence remains limited and inconsistent.

Many studies failed to report ICU LOS as a primary outcome or lacked group-specific mean and standard deviation data. In several cases, ICU LOS was embedded within multivariable regression models or described solely for the OSA cohort, precluding direct comparison. The absence of standardized reporting criteria further contributes to the interpretative challenge.

Despite these limitations, a qualitative synthesis suggests a possible trend toward prolonged ICU stay in patients with OSA, particularly those with moderate-to-severe disease. This may reflect increased perioperative complications, extended ventilatory support requirements, or heightened monitoring needs.

Given the high costs and resource demands associated with ICU care, future studies should aim to quantify the impact of OSA on ICU LOS using uniform definitions and robust statistical models. Such analyses would enhance our understanding of the healthcare burden imposed by untreated or unrecognized sleep-disordered breathing in cardiac surgical populations.

## 4. Discussion

This systematic review provides a comprehensive synthesis of the current literature examining the association between obstructive sleep apnea and postoperative outcomes in cardiac surgery. Seventeen primary studies were included in the qualitative synthesis, with four additional studies contributing data to meta-analyses of specific outcomes. Collectively, these studies offer robust evidence that OSA constitutes a significant and potentially modifiable perioperative risk factor in patients undergoing procedures such as coronary artery bypass grafting (CABG), aortic valve replacement, and mitral valve surgery [47,48,49,50,51,52,54,55,56,58,59,60,61,62,63,64,65,66,67,68,72].

Among the prospective studies, the prevalence of OSA exceeded 60%, with moderate-to-severe disease (AHI > 15) affecting over 70% of patients. These figures underscore the underappreciated diagnostic burden of OSA when active screening or polysomnography is implemented. In contrast, retrospective datasets relying on administrative codes reported substantially lower prevalence estimates (~22%), highlighting the diagnostic gap associated with passive or indirect case identification [51,52,54,55,56,62,63,64,65,66,68,72]

The findings also confirm that OSA is associated with a higher risk of several clinically meaningful outcomes. Meta-analyses demonstrated increased odds for postoperative atrial fibrillation (OR = 2.44), major adverse cardiac and cerebrovascular events (MACCE) (OR = 2.06), acute kidney injury (AKI) (OR = 2.24), and respiratory complications (OR = 1.15). Hospital length of stay was also prolonged (SMD = 0.62), and pneumonia risk was marginally increased (OR = 1.07). In contrast, findings regarding stroke, ICU length of stay, and mortality were less consistent and did not meet statistical thresholds across pooled analyses [47,48,49,50,55].

These results support the assertion that OSA exerts a clinically significant impact on perioperative morbidity. Importantly, many of the complications associated with OSA are potentially preventable or modifiable through preoperative risk stratification, use of positive airway pressure (PAP) therapy, intraoperative management strategies, and enhanced postoperative monitoring [73,74,75,76].

This review also highlights the need for standardized diagnostic criteria and consistent reporting of sleep metrics and surgical outcomes. Prospective studies utilizing validated tools, objective endpoints, and multicenter designs are essential to further elucidate the mechanisms by which OSA contributes to adverse surgical outcomes and to assess the efficacy of targeted interventions.

### 4.1. Mechanistic Insights

The pathophysiological mechanisms underlying the association between obstructive sleep apnea and adverse surgical outcomes are multifactorial and increasingly well characterized. OSA is typified by repetitive upper airway collapse during sleep, resulting in intermittent hypoxia, hypercapnia, and frequent arousals from sleep. These physiological disturbances trigger a cascade of systemic responses that negatively influence perioperative stability and postoperative recovery [77,78,79].

Intermittent hypoxia activates sympathetic nervous system pathways, leading to elevated catecholamine levels, increased blood pressure, and heightened myocardial oxygen demand [18,80,81,82,83,84]. These changes contribute to arrhythmogenicity and may explain the observed association between OSA and postoperative atrial fibrillation [85]. Hypoxia also induces oxidative stress and systemic inflammation, both of which have been implicated in endothelial dysfunction and plaque instability, potentially elevating the risk of ischemic events and acute kidney injury [86,87,88,89,90,91,92].

In terms of respiratory complications, OSA is associated with impaired ventilatory control, increased upper airway resistance, and heightened opioid sensitivity. These factors elevate the risk of postoperative hypoventilation, prolonged intubation, and pneumonia. Additionally, the disruption of normal sleep architecture may impair immune function and tissue healing [93,94,95,96,97,98,99,100,101].

Neurohumoral activation, endothelial damage, and microvascular dysfunction are other plausible mechanisms by which OSA may exacerbate perioperative morbidity. These systemic effects are particularly relevant in the context of cardiac surgery, where inflammatory burden, ischemia–reperfusion injury, and hemodynamic shifts are already pronounced [102,103,104,105,106,107,108,109].

Collectively, these mechanisms offer a plausible explanation for the range of complications observed in patients with untreated or inadequately managed OSA undergoing cardiac surgery [43,48,58,110,111]. Further mechanistic studies, including biomarker-based and hemodynamic monitoring investigations, are warranted to refine our understanding and inform targeted therapeutic strategies [112,113].

### 4.2. Comparison to the Previous Literature

The findings of this systematic review are broadly consistent with earlier research highlighting the adverse impact of obstructive sleep apnea on perioperative outcomes in cardiac surgery. Prior studies have identified OSA as a risk factor for postoperative complications, particularly atrial fibrillation, respiratory insufficiency, and prolonged hospitalization [114,115].

Meta-analyses by Wang et al. (2020) [47] and Nagappa et al. (2017) [48] similarly reported elevated odds for major adverse cardiac and cerebrovascular events and atrial fibrillation in OSA populations. The present review confirms these findings and extends them by disaggregating outcomes such as acute kidney injury, pneumonia, and length of stay. This additional granularity strengthens the case for routine OSA screening and perioperative management.

In contrast, several studies including Patel et al. (2018) [49] and Javaherforooshzadeh et al. (2022) [50] have underscored methodological inconsistencies in the literature, particularly concerning OSA diagnosis and outcome reporting. This review acknowledges these limitations and attempts to address them by stratifying findings by study design, screening method, and population characteristics.

Notably, the association between OSA and mortality or stroke was less pronounced in the current analysis compared to earlier reports [72,116,117,118,119]. This discrepancy may reflect improved perioperative care, advancements in surgical techniques, and increased awareness of OSA as a comorbidity. Alternatively, it may be attributable to methodological limitations such as small sample sizes, low event rates, and inadequate follow-up durations.

Overall, this review supports and extends the existing literature while emphasizing the need for greater standardization in future research. The convergence of findings across studies reinforces the role of OSA as a clinically meaningful, yet often underappreciated, risk factor in the cardiac surgical setting.

### 4.3. Clinical Implications

The findings of this review carry important implications for the perioperative management of patients undergoing cardiac surgery. Given the high prevalence of obstructive sleep apnea in this population and its consistent association with adverse outcomes, routine preoperative screening should be strongly considered. Instruments such as the STOP-Bang and STOP-BAG2 questionnaires offer pragmatic and clinically validated tools for identifying high-risk individuals, particularly when resources for polysomnography are limited [38,120,121]. Their integration into anesthesia preoperative evaluations or cardiology-driven risk stratification pathways could improve early detection and perioperative planning.

For patients identified with moderate-to-severe OSA, the initiation or reinforcement of continuous positive airway pressure therapy prior to surgery may mitigate risks related to arrhythmias, respiratory complications, and prolonged recovery. However, a significant limitation of the current evidence base is the paucity of data on CPAP adherence and perioperative implementation. Most studies included in this review did not document whether patients received or complied with OSA treatment, limiting the ability to determine the direct effect of intervention on surgical outcomes. This gap is particularly relevant given that therapy adherence likely mediates many of the complications observed in OSA cohorts [122].

In the postoperative setting, enhanced respiratory monitoring, early mobilization, opioid-sparing analgesia, and careful fluid management may be particularly beneficial in OSA patients [123,124]. Institutions should consider embedding OSA screening within enhanced recovery after surgery (ERAS) protocols or developing formal perioperative care bundles. Importantly, the implementation of such pathways will require interdisciplinary coordination among anesthesiologists, sleep medicine specialists, cardiologists, and surgical teams. The modifiable nature of OSA and the feasibility of targeted interventions make it a compelling focus for improving outcomes in high-risk surgical populations [125].

**Figure 13:**
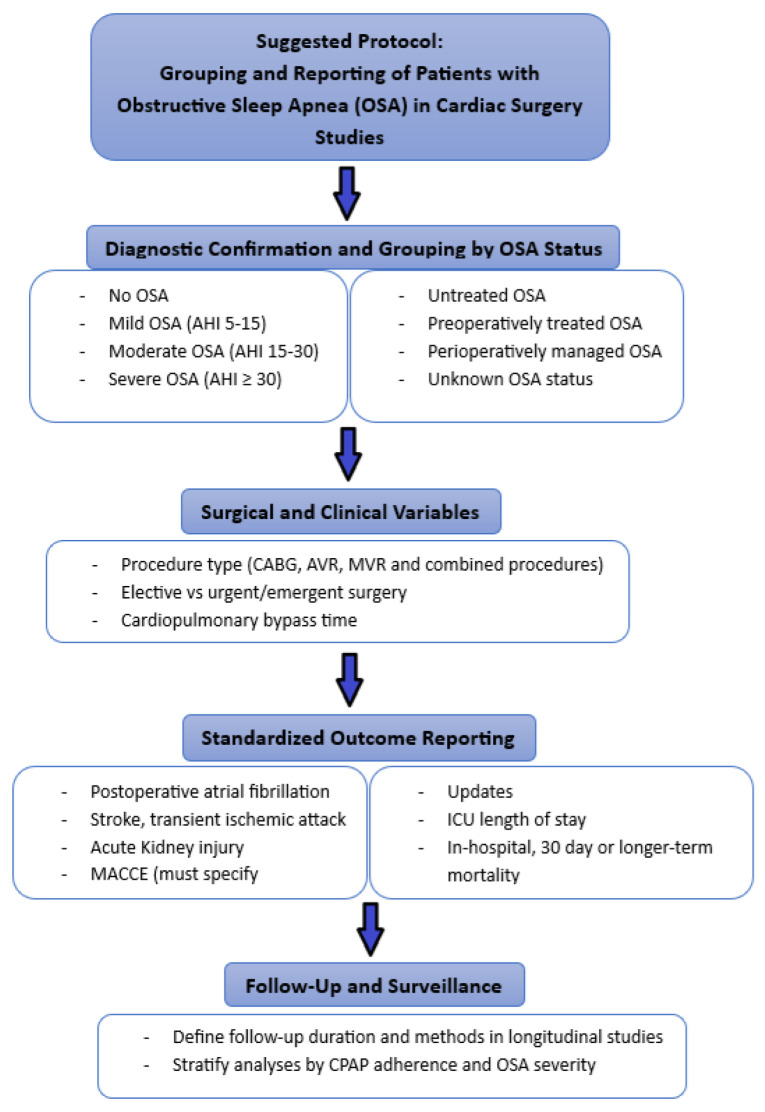
Suggested protocol for the grouping and reporting of patients with obstructive sleep apnea in cardiac surgery studies.

To promote standardization in future research and enhance the comparability of findings across studies, we propose a structured framework for the classification, analysis, and reporting of patients with OSA in the context of cardiac surgery. 

(Figure 13). This protocol includes stratification by OSA severity and treatment status, incorporation of key surgical and clinical variables, standardized outcome definitions, and follow-up parameters. Adopting such an approach may facilitate meta-analytic synthesis, improve reporting transparency, and ultimately support more tailored perioperative care strategies.

### 4.4. Limitations and Future Directions

Despite the comprehensive nature of this systematic review and meta-analysis, several limitations warrant consideration. First, substantial heterogeneity was present among the included studies, particularly with respect to the methods used for diagnosing obstructive sleep apnea. While some studies employed gold-standard polysomnography, others relied on screening questionnaires or administrative coding, introducing potential classification bias and limiting the comparability of findings. Moreover, inconsistencies in the application of apnea–hypopnea index thresholds further complicate direct comparisons across studies.

Second, many studies lacked granular clinical data, including OSA severity, continuous positive airway pressure adherence, and the timing of perioperative interventions. These omissions hindered efforts to stratify patients by risk level and assess the true impact of therapeutic measures. Given that CPAP use is a key modifiable factor, its underreporting represents a critical gap in the current evidence base.

Third, several important clinical outcomes—such as intensive care unit length of stay, stroke, and mortality—were either inconsistently reported or underpowered, precluding robust meta-analytic synthesis. The absence of standardized outcome definitions and limited subgroup analyses further constrained interpretability. Additionally, the inclusion of studies with overlapping patient populations in both qualitative and quantitative syntheses may have introduced an element of duplication bias, despite efforts to avoid this.

Fourth, the predominance of observational study designs limits causal inference. Confoundments by comorbidities such as obesity, hypertension, and heart failure may have influenced outcome estimates, even in studies that attempted multivariable adjustment.

Future research should prioritize large-scale, prospective, multicenter trials with clearly defined diagnostic criteria and standardized outcome reporting. Particular attention should be paid to quantifying CPAP adherence and perioperative implementation, as well as exploring the impact of OSA treatment on clinically meaningful endpoints. Additionally, efforts to harmonize definitions for perioperative complications and integrate sleep-disordered breathing into risk stratification models are necessary to translate observational findings into actionable clinical practice.

The narrative risk of bias assessment, as outlined in the PROSPERO protocol, identified potential sources of bias related to selection criteria, diagnostic heterogeneity, and outcome reporting. These limitations should be considered when interpreting the findings, particularly regarding the strength of evidence for pooled effect estimates.

### 4.5. Gaps in the Current Literature on OSA and Cardiac Surgery

Despite growing evidence linking obstructive sleep apnea (OSA) to adverse cardiovascular and surgical outcomes, several key gaps persist in the current literature as follows:

- “Lack of uniform OSA diagnosis and severity stratification”: Many studies rely on administrative codes, screening questionnaires (e.g., STOP-Bang), or self-reported history rather than gold-standard polysomnography. The inconsistent application of apnea-hypopnea index (AHI) thresholds—such as ≥5, ≥15, or ≥30 events per hour—limits comparability across studies and hinders pooled data analysis.
- “Heterogeneity in outcome reporting”: Definitions and metrics for outcomes such as atrial fibrillation, myocardial infarction, acute kidney injury (AKI), and ICU length of stay vary widely. This inconsistency impairs direct comparisons and undermines the strength of cumulative evidence.
- “Limited focus on treatment effects”: Few studies explicitly evaluate the impact of therapeutic interventions, such as preoperative or perioperative CPAP use. The modifiability of OSA-related risk remains largely speculative in the absence of controlled trials.
- “Dominance of underpowered or retrospective designs”: A majority of studies are single-center and observational, limiting statistical power and increasing susceptibility to selection and information bias. This contributes to equivocal findings for key endpoints such as stroke and mortality.
- “Insufficient integration into perioperative protocols”: Despite strong evidence of risk, OSA is infrequently incorporated into formal risk assessment tools or clinical pathways for cardiac surgery. Current guidelines do not include OSA as a standard component of preoperative evaluation, highlighting a disconnect between evidence and practice.

Addressing these gaps through standardized diagnostic protocols, prospective multicenter designs, and trials evaluating therapeutic interventions will be essential to fully realize the clinical relevance of OSA in cardiac surgery.

## 5. Conclusions

This systematic review and meta-analysis demonstrate that obstructive sleep apnea is a prevalent and clinically impactful comorbidity in patients undergoing cardiac surgery. The presence of OSA was consistently associated with an increased risk of major perioperative complications, including atrial fibrillation, major adverse cardiac and cerebrovascular events, acute kidney injury, respiratory complications, and prolonged hospital length of stay. Although associations with stroke, ICU length of stay, and postoperative mortality were less consistent, the overall pattern of findings suggests a substantive and potentially modifiable risk profile.

Importantly, many of the adverse outcomes linked to OSA may be mitigated through early identification and targeted perioperative management, such as the use of continuous positive airway pressure therapy. However, current evidence is limited by the heterogeneity in diagnostic methods, underreporting of treatment adherence, and inconsistent outcome definitions.

These findings underscore the need for routine OSA screening as part of preoperative risk assessment in cardiac surgery candidates. Future prospective, multicenter trials incorporating standardized diagnostic criteria, clearly defined endpoints, and interventional strategies are essential to determine the clinical benefit of structured OSA management. Addressing these gaps represents a critical opportunity to improve perioperative outcomes and inform evidence-based guidelines for this high-risk surgical population.

## Figures and Tables

**Table 1:** Detailed overview of study characteristics like study design, sample size, and population demographics.

Article	Study Design	Sample Size	Geographic Location
Foldvary-Schaefer et al., 2015 [51]	Prospective	107	USA
Gali et al., 2020 [58]	Retrospective	8612	USA
Wolf et al., 2021 [59]	Retrospective	12,505	USA
Wong et al., 2015 [60]	Retrospective	545	Canada
Uchôa et al., 2015 [52]	Prospective	67	Brazil
Koo et al., 2020 [53]	Prospective	1007	South Korea
Tafelmeier et al., 2020 [54]	Prospective	250	Germany
Liamsombut et al., 2021 [55]	Prospective	107	Thailand
Krishnasamy et al., 2019 [61]	Retrospective	101	Australia
Ding et al., 2016 [56]	Prospective	290	China
Keymel et al., 2015 [62]	Prospective	48	Germany
Oldham et al., 2024 [63]	Prospective	46	USA
Fan et al., 2021 [64]	Prospective	147	China
Peker et al., 2022 [65]	Prospective	147	Sweden
Teo et al., 2021 [66]	Prospective	1007	Singapore
Feng et al., 2019 [67]	Retrospective	506,604	USA
Sezai et al., 2016 [68]	Prospective	1005	Japan

**Table 2:** Key findings from the retrospective studies, including OSA prevalence, demographic patterns, and body mass index profiles.

Total Patients	OSA (n, %)	CSA (n, %)	Age (OSA)	M (n, %)	F (n, %)	BMI (OSA)
8612	2636 (30.6%)	ns	65.3	2001 (75.9%)	635 (24.1%)	33.8
12,505	1555 (12.4%)	ns	65.4	1245 (80.1%)	310 (19.9%)	34.8
545	72 (13.2%)	ns	67.8 ± 10.2	57 (79.2%)	15 (20.8%)	ns
101	62 (61.4%)	ns	60 ± 8	49 (79.0%)	15 (24.2%)	ns
506,604	32,545 (6.4%)	ns	64.5 ± 10.5	24,977 (76.7%)	7568 (23.3%)	ns
528,367	36,870 (6.97%)	ns	64.6	28,329 (76.83%)	8543 (23.17%)	34.3

OSA—Obstructive sleep apnea; CSA—Central sleep apnea; M—Male; F—Female, BMI—Body mass Index.

**Table 3:** Concise summary of each tool’s strengths, weaknesses, and clinical verdict.

Tool	Strengths	Weaknesses	Verdict
Epworth Sleepiness Scale	Easy to administer	Poor sensitivity/specificity; symptom-based	Not suitable
Berlin Questionnaire	Familiar tool	Weak performance in high-comorbidity groups	Limited utility
STOP	Simple, high-yield question (observed apnea)	Low specificity, subjective symptoms	Moderate utility
STOP-Bang	Includes objective risk factors	Not tested directly in all studies	Promising, needs validation
STOP-BAG2	Strongest overall performance	Retrospective use; not widely implemented yet	Best current option
SA/SDQ (SAS Scale)	Broad scope; modest accuracy in men	Lengthy; impractical clinically	Conditional use

STOP—Snoring, Tiredness, Observed apnea, high blood Pressure—a 4-item screening tool for obstructive sleep apnea (OSA); STOP-Bang—STOP questionnaire plus Body mass index, Age, Neck circumference, and Gender—an 8-item screening tool for OSA; STOP-BAG2—STOP-Bang plus additional items including history of Gastroesophageal reflux disease (GERD) and Glucose intolerance (diabetes), forming a 10-item screening tool for OSA.

**Table 4:** Summary of key studies evaluating tool performance in cardiac surgery populations [47,50,51,55,61].

Article	Tools Used	Associated with OSA Diagnosis	Performance Metrics
Foldvary-Schaefer et al., 2015 [51]	Epworth, Berlin, STOP, SA/SDQ	No clear correlation (ESS poor; STOP-BAG2 best)	STOP-BAG2 AUC = 0.66; STOP AUC = 0.61; ESS AUC~0.52
Javaherforooshzadeh et al., 2022 [50]	STOP-Bang	Yes (high STOP-Bang risk predicted complications)	Not reported as AUC; regression showed significance (*p* = 0.002)
Krishnasamy et al., 2019 [61]	STOP-Bang, ApneaLink portable monitor	Yes (portable monitor validated); STOP-Bang sensitivity 75.8%	STOP-Bang: Sensitivity 75.8%; mean STOP-Bang OSA vs. non-OSA: 3.4 vs. 3.0
Wang et al., 2020 [47], (Meta-analysis)	Mixed (some STOP-Bang, Berlin, PSG)	Yes; confirmed in most included studies	Not separated per tool; pooled OR for MACCE = 1.97
Liamsombut et al., 2021 [55]	STOP, STOP-BAG2, SA/SDQ, Berlin, ESS	Partial; STOP-BAG2 best, ESS and Berlin poor	STOP-BAG2 AUC = 0.66; ESS AUC~0.52

STOP-BAG2 and STOP-Bang emerge as the most promising tools for OSA screening in cardiac surgery populations, while ESS consistently demonstrates suboptimal diagnostic capacity. Future research should prioritize tool-specific meta-analyses using pooled individual patient-level data. STOP—Snoring, Tiredness, Observed apnea, high blood Pressure—a 4-item screening tool for obstructive sleep apnea (OSA); SA/SDQ—Sleep Apnea/Sleep-Disordered Breathing Questionnaire—a tool used to assess symptoms related to sleep-disordered breathing; AUC—Area Under the Curve—a measure of diagnostic test accuracy; PSG—Polysomnography; OR—Odds Ratio; MACCE—Major Adverse Cardiac and Cerebrovascular Events

## Data Availability

The protocol for this review is publicly accessible via the PROSPERO database under the registration number CRD420251049574. The original contributions presented in this study are included in the article. Further inquiries can be directed to the corresponding authors.

## Abbreviations

The following abbreviations are used in this manuscript:

AHI	Apnea-Hypopnea Index
AKI	Acute Kidney Injury
ASA	American Society of Anesthesiologists
AUC	Area Under the Curve
AVR	Aortic Valve Replacement
BMI	Body Mass Index
CABG	Coronary Artery Bypass Grafting
CI	Confidence Interval
CPAP	Continuous Positive Airway Pressure
CSA	Central Sleep Apnea
ERAS	Enhanced Recovery After Surgery
ESS	Epworth Sleepiness Scale
F	Female
hs-CRP	High-Sensitivity C-Reactive Protein
ICD	International Classification of Diseases
ICU	Intensive Care Unit
LOS	Length of Stay
M	Male
MACCE	Major Adverse Cardiac and Cerebrovascular Events
MI	Myocardial Infarction
MVR	Mitral Valve Repair or Replacement
ns	Not Specified
OR	Odds Ratios
OSA	Obstructive Sleep Apnea
PAP	Positive Airway Pressure
POAF	Postoperative Atrial Fibrillation
PRISMA	Preferred Reporting Items for Systematic Reviews and Meta-Analyses
PSG	Polysomnography
SA-SDQ	Sleep Apnea-Specific Distress Questionnaire
SMD	Standardized Mean Difference
STOP-BAG2	Screening Tool for Obstructive Sleep Apnea—Modified Version 2
STOP-Bang	Snoring, Tiredness, Observed Apnea, Blood Pressure, BMI, Age, Neck circumference, Gender
TIA	Transient Ischemic Attack

## Appendix A

Overview of Recent Publications on Sleep Apnea associated with Cardiac Surgery (2015–2025).

**Number**	**Article Name**	**Study Type**	**Number of Patients**	**Age (Years) OSA**	**Male OSA**	**Female OSA**
1	Foldvary-Schaefer et al., 2015 [51]	Prospective	107	69.7 ± 12.5	ns	ns
2	Gali et al., 2020 [58]	Retrospective	8612	65.3	2001	635
3	Wolf et al., 2021 [59]	Retrospective	12,505	65.4	1245	310
4	Nagappa et al., 2017 [48]					
5	Wong et al., 2015 [60]	Retrospective	545	67.8 ± 10.2	57	15
6	Uchôa et al., 2015 [52]	Prospective	67	59.0 ± 7.9	31	6
7	Koo et al., 2020 [53]	Prospective	1007	62	442	71
8	Tafelmeier et al., 2020 [54]	Prospective	250	67.9 ± 7.7	112	15
9	Liamsombut et al., 2021 [55]	Prospective	107	69.7 ± 12.5	33	18
10	Javaherforooshzadeh et al., 2022 [50]					
11	Krishnasamy et al., 2019 [61]	Retrospective	101	60 ± 8	49	15
12	Wang et al. (2020) [47]					
13	Patel et al. (2018) [49]					
14	Ding et al., 2016 [56]	Prospective	290	55.20 ± 9.05	61	54
15	Keymel et al., 2015 [62]	Prospective	48	81 ± 8	ns	ns
16	Oldham et al., 2024 [63]	Prospective	46	67.1	31	14
17	Fan et al., 2021 [64]	Prospective	147	62.9 ± 8.2	47	15
18	Peker et al., 2022 [65]	Prospective	147	66.5 ± 7.5	ns	ns
19	Teo et al., 2021 [66]	Prospective	1007	62	442	71
20	Feng et al., 2019 [67]	Retrospective	506,604	64.5 ± 10.5	24,977	7568
21	Sezai et al., 2016 [68]	Prospective	1005	69.3 ± 10.3	519	259

**Number**	**Epworth Sleeping Scale**	**Questionnaires**	**Berlin**	**STOP**	**STOPBANG2**
1	yes	yes	yes	ns	ns
2	ns	ns	ns	ns	ns
3	ns	ns	ns	ns	ns
4					
5	ns	ns	ns	ns	ns
6	ns	ns	ns	ns	ns
7	yes > 10 in 69 patients	yes	239 patients with high risk	ns	ns
8	ns	ns	ns	ns	ns
9	yes > 10 in 35 patients	yes	49 patients with high risk	≥2 in 72 de patients	It differentiated patients with AHI greater or lower than 15.
10					
11	yes > 10 in 4 patients	yes	ns	ns	≥3: 47 of the OSA group
12					
13					
14	ns	ns	ns	ns	ns
15	ns	ns	ns	ns	ns
16	yes > 10 in 7 patients	Pittsburgh Sleep Quality Index > 5 la 34 patients, Patient Health Questionnaire-9	ns	ns	ns
17	ns	ns	ns	ns	ns
18	yes > 10 in 18 patients	ns	ns	ns	ns
19	yes < 10 in all groups	yes	yes	ns	ns
20	ns	ns	ns	ns	ns
21	ns	ns	ns	ns	ns

**Number**	**SAS Scale of Sleep Disorder Questionnaire**	**OSA**	**CSA**	**AHI < 5**	**AHI > 5**	**AHI > 15**	**AHI > 30**	**Mean AHI**	**SpO2 < 90%**	**Pre-Op PAP Therapy**	**BMI OSA**
1	ns	79	ns	28	28	22	29	ns	ns	no	26.8 ± 6.4
2	ns	2636	ns	ns	ns	ns	ns	ns	ns	ns	33.8
3	ns	1555	ns	ns	ns	ns	ns	ns	ns	ns	34.8
4											
5	ns	72	ns	ns	ns	ns	ns	ns	ns	32	ns
6	ns	37	ns	ns	ns	37	ns	36.4 ± 18.8	28	ns	29.1 ± 4.4
7	ns	513	ns	ns	ns	513	ns	30.1	385	ns	25.9
8	ns	59	68	ns	ns	127	ns	15.2	50	ns	30.1 ± 4.6
9	≥ 32 for women and ≥ 36 for men corelated with OSA diagnosis	77	ns	30	26	51	ns	ns	ns	no	26.5 ± 6.6
10											
11	ns	62	ns	39	35	13	14	25.8 ± 4.3	ns	no	ns
12											
13											
14	ns	54	61	175	115	ns	ns	ns	ns	no	23.85 ± 3.7
15	ns	36	1	11	37	18	ns	ns	28	no	26 ± 5
16	ns	39	6	1	15	18	12	ns	35	no	32.4
17	ns	62	ns	ns	ns	62	ns	26.2	ns	no	25.2 ± 3.7
18	ns	141	ns	6	21	53	67	ns	37	ns	28.0 ± 4.5
19	ns	800	ns	207	287	254	259	ns	ns	no	ns
20	ns	32,545	ns	ns	ns	ns	ns	ns	ns	ns	ns
21	ns	778	ns	227	361	260	157	ns		no	24.3 ± 3.8

**Number**	**BMI CSA**	**BMI AHI < 15**	**BMI AHI > 15**	**EF in AHI > 15**	**EF < 30**	**EF: 30–50**	**EF < 40**	**EF < 45**	**EF > 45**	**EF: 50**	**EF < 55**	**Mean EF**
1	ns	26.3 ± 6.8	26.8 ± 6.4	ns				72	26	ns	ns	43.6 ± 15.4
2	ns	ns	ns	ns				ns	ns	ns	ns	ns
3	ns	ns	ns	ns	ns	ns	153	ns	ns	ns	ns	ns
4												
5	ns	ns	ns	ns	ns	ns	ns	ns	ns	ns	18	ns
6	ns	ns	29.1 ± 4.4	ns	ns	ns	ns	ns	ns	ns	ns	49.2 ± 14.2
7	ns	ns	25.9	ns	84	158	ns	ns	ns	223	ns	ns
8	28.5 ± 4.1	ns	ns	ns	ns	ns	ns	ns	ns	ns	41	51.8 ± 13
9	ns	26.3 ± 6.8	26.8 ± 6.4	43.6 ± 15.4	ns	ns	ns	ns	ns	ns	ns	ns
10												
11	ns	ns	ns	ns	ns	ns	ns	ns	ns	ns	ns	ns
12												
13												
14	23.32 ± 3.04	ns	ns	ns	ns	ns	ns	ns	ns	ns	ns	58.97 ± 8.27
15	ns	26 ± 6	26 ± 5	52 ± 10	ns	ns	ns	ns	ns	ns	ns	ns
16	ns	27.4	32.4	ns	ns	ns	ns					
17	ns	ns	25.2 ± 3.7	56.8 ± 10.2	ns	ns	ns	ns	ns	ns	ns	ns
18	ns	ns	ns	ns	ns	ns	ns	ns	ns	ns	ns	ns
19	ns	ns	ns	ns	ns	ns	ns	ns	ns	ns	ns	ns
20	ns	ns	ns	ns	ns	ns	ns	ns	ns	ns	ns	ns
21	ns	22.3 ± 3.2	23.2 ± 3.5	ns	ns	ns	ns	ns	ns	ns	ns	ns

**Number**	**Questionnaire Relevance**	**Post Op Arrhythmia OSA**	**Post Op Arrhythmia AHI < 15**	**Post Op Arrhythmia AHI > 15**	**Post Op Arrhythmia AHI > 30**	**Post Op AF OSA**	**Post Op AF AHI < 15**	**Post Op AF AHI > 15**
1	ns	ns	26	28	19	ns	ns	ns
2	ns	ns	ns	ns	ns	825	ns	ns
3	ns	ns	ns	ns	ns	482	ns	ns
4								
5	ns	ns	ns	ns	ns	48	ns	ns
6	ns	16	ns	16	ns	10	ns	10
7	ns	ns	ns	ns	ns	ns	84	ns
8	ns	ns	ns	ns	ns	ns	ns	ns
9	Suboptimal performance. STOPBANG2 had better performance characteristics		ns	ns	ns	ns	ns	ns
10								
11	No. STOP-BANG had a higher sensitivity of 75.8%	ns	ns	ns	ns	7	ns	ns
12								
13								
14	ns	46	ns	ns	ns	19 OSA and 45 CSA. Total: 64	ns	ns
15	no	ns	ns	ns	ns	ns	ns	ns
16								
17	ns	ns	ns	ns	ns	ns	ns	ns
18	ns	ns	ns	ns	ns	46	7	17
19	no	ns	ns	ns	ns	ns	ns	ns
20	ns	ns	ns	ns	ns	1967	ns	ns
21	ns	ns	ns	ns	ns	190	43	74

**Number**	**Post Op AF AHI > 30**	**Intubation Time (h) OSA**	**Intubation Time (h) CSA**	**Intubation Time (h) AHI < 15**	**Intubation Time (h) AHI > 15**	**Intubation Time (h) AHI > 30**	**Length of Stay (ICU) (Days) OSA**	**Length of Stay (ICU) (Days) AHI < 15**
1	ns	ns	ns	14.5	16.6	18	ns	2
2	ns	24.4	ns	ns	ns	ns	2.22	ns
3	ns	6.6	ns	ns	ns	ns	ns	ns
4			ns					
5	ns	ns	ns	ns	ns	ns	ns	ns
6	ns	ns	ns	ns	ns	ns	ns	ns
7	ns	ns	ns	ns	ns	ns	ns	ns
8	ns	12	17	ns	ns	ns	4.5	ns
9	ns	ns	ns	ns	ns	ns	ns	ns
10								
11	ns	ns	ns	ns	ns	ns	5.5 ± 5.1	ns
12								
13								
14	ns	17	17	ns	ns	ns	1.5	ns
15	ns	ns	ns	ns	ns	ns	ns	ns
16								
17	ns	ns	ns	ns	ns	ns	ns	ns
18	22	ns	ns	ns	ns	ns	ns	ns
19	ns	ns	ns	ns	ns	ns	ns	ns
20	ns	ns	ns	ns	ns	ns	ns	ns
21	73	ns	ns	ns	ns	ns	ns	ns

**Number**	**Length of Stay (ICU) (Days) AHI > 15**	**Length of Stay (ICU) (Days) AHI > 30**	**ICU Readmision OSA**	**ICU Readmision AHI < 15**	**ICU Readmision AHI > 15**	**ICU Readmision AHI > 30**	**AKIN OSA**	**IABP OSA**	**Respiratory Insufficiency OSA**
1	2.5	3	ns	6	9	7	ns	ns	ns
2	ns	ns	141	ns	ns	ns	663	ns	ns
3	ns	ns	84	ns	ns	ns	36	54	ns
4									
5	ns	ns	ns	ns	ns	ns	5	ns	ns
6	ns	ns	ns	ns	ns	ns	ns	ns	ns
7	ns	ns	ns	ns	ns	ns	ns	ns	ns
8	ns	ns	ns	ns	ns	ns	ns	ns	ns
9	ns	ns	ns	ns	ns	ns	ns	ns	ns
10									
11	ns	ns	1	ns	ns	ns	ns	ns	ns
12									
13									
14	ns	ns	ns	ns	ns	ns	ns	ns	20
15	ns	ns	ns	ns	ns	ns	ns	ns	ns
16									
17	ns	ns	ns	ns	ns	ns	ns	ns	ns
18	ns	ns	ns	ns	ns	ns	ns	ns	ns
19	ns	ns	ns	ns	ns	ns	ns	ns	ns
20	ns	ns	ns	ns	ns	ns	ns	ns	ns
21	ns	ns	ns	ns	ns	ns	ns	ns	ns

**Number**	**Respiratory Insufficiency CSA**	**Pneumonia OSA**	**Hospital Mortality OSA**	**Hospital Mortality AHI < 15**	**Hospital Mortality AHI > 15**	**Hospital Mortality AHI > 30**	**Follow-Up (Years)**	**Cardiovascular Deaths**
1	ns	ns	ns	4	5	4	ns	ns
2	ns	76	51	ns	ns	ns	ns	ns
3	ns	59	35	ns	ns	ns	ns	ns
4							ns	ns
5	ns	ns	ns	ns	ns	ns	ns	ns
6	ns	ns	ns	ns	ns	ns	4.5	3
7	ns	ns	ns	ns	ns	ns	2.1	18
8	ns	15	4	ns	ns	ns	ns	ns
9	ns	ns	ns	ns	ns	ns	ns	ns
10								
11	ns	3	3	0	1	2	ns	ns
12								
13								
14	17	ns	ns	ns	ns	ns	ns	ns
15	ns	ns	ns	ns	ns	ns	ns	ns
16								
17	ns	ns	ns	ns	ns	ns	ns	ns
18	ns	ns	ns	ns	ns	ns	ns	ns
19	ns	ns	ns	ns	ns	ns	ns	ns
20	ns	624	729	ns	ns	ns	7	ns
21	ns	ns	ns	ns	ns	ns	ns	ns

**Number**	**Non-Fatal Myocardial Infarction**	**Non-Fatal Strokes**	**Unplanned Revascularization**	**MACCE Incidence %**
1	ns	ns	ns	ns
2	ns	ns	ns	ns
3	ns	ns	ns	ns
4	ns	ns	ns	ns
5	ns	ns	ns	ns
6	8	3	8	49
7	22	26	18	14.7
8	ns	ns	ns	ns
9	ns	ns	ns	ns
10				
11	1	1	ns	ns
12				
13				
14	ns	ns	ns	ns
15	ns	ns	ns	ns
16				
17	17	ns	ns	ns
18	ns	ns	ns	ns
19	ns	ns	ns	ns
20	8337	4247	ns	ns
21	ns	ns	ns	ns

## Appendix B

Prisma flow diagram summarizing the identification, screening, eligibility assessment, and inclusion of studies in the systematic review. The diagram adheres to the PRISMA 2020 guidelines and outlines the number of records identified through database searching, screened, assessed for eligibility, excluded with reasons, and ultimately included in qualitative and quantitative syntheses.

## Appendix C

Summarizes the aggregate data from prospective studies, reflecting high diagnostic yield, elevated BMI profiles, and male predominance.

**Article**	**Total Patients**	**OSA (n, %)**	**CSA (n, %)**	**Age (OSA)**	**M (n, %)**	**F (n, %)**	**AHI < 5 (n, %)**	**AHI > 5 (n, %)**	**AHI > 15 (n, %)**	**AHI > 30 (n, %)**	**BMI (OSA)**
Foldvary-Schaefer et al., 2015 [51]	107	79 (73.8%)	ns	69.7 ± 12.5	ns	ns	28 (35.4%)	28 (35.4%)	22 (27.8%)	29 (36.7%)	26.8 ± 6.4
Uchôa et al., 2015 [52]	67	37 (55.2%)	ns	59.0 ± 7.9	31 (83.8%)	6 (16.2%)	ns	ns	37 (100.0%)	ns	29.1 ± 4.4
Koo et al., 2020 [53]	1007	513 (50.9%)	ns	62	442 (86.2%)	71 (13.8%)	ns	ns	513 (100.0%)	ns	25.9
Tafelmeier et al., 2020 [54]	250	59 (23.6%)	68 (27.2%)	67.9 ± 7.7	112 (88.2)	15 (25.4%)	ns	ns	127 (215.3%)	ns	30.1 ± 4.6
Liamsombut et al., 2021 [55]	107	77 (72.0%)	ns	69.7 ± 12.5	33 (42.9%)	18 (23.4%)	30 (39.0%)	26 (33.8%)	51 (66.2%)	ns	26.5 ± 6.6
Ding et al., 2016 [56]	290	54 (18.6%)	61 (21.0%)	55.20 ± 9.05	61 (70,15%)	54 (62.1%)	175 (60.34%)	115 (39.65%)	ns	ns	23.85 ± 3.7
Keymel et al., 2015 [62]	48	36 (75.0%)	1 (2.1%)	81 ± 8	ns	ns	11 (30.6%)	37 (102.8%)	18 (50.0%)	ns	26 ± 5
Oldham et al., 2024 [63]	46	39 (84.8%)	6 (13.0%)	67.1	31 (79.5%)	14 (35.9%)	1 (2.6%)	15 (38.5%)	18 (46.2%)	12 (30.8%)	32.4
Fan et al., 2021 [64]	147	62 (42.2%)	ns	62.9 ± 8.2	47 (75.8%)	15 (24.2%)	ns	ns	62 (100.0%)	ns	25.2 ± 3.7
Peker et al., 2022 [65]	147	141 (95.9%)	ns	66.5 ± 7.5	ns	ns	6 (4.3%)	21 (14.9%)	53 (37.6%)	67 (47.5%)	28.0 ± 4.5
Teo et al., 2021 [66]	1007	800 (79.4%)	ns	62	442 (55.2%)	71 (8.9%)	207 (25.9%)	287 (35.9%)	254 (31.8%)	259 (32.4%)	ns
Sezai et al., 2016 [68]	1005	778 (77.4%)	ns	69.3 ± 10.3	519 (66.7%)	259 (33.3%)	227 (29.2%)	361 (46.4%)	260 (33.4%)	157 (20.2%)	24.3 ± 3.8
Total	4228	2675 (63.3%)	136 (3.2%)	66.0	1718 (61.1%)	523 (18.6%)	685 (16.2%)	890 (31.7%)	1415 (50.3%)	524 (18.6%)	29.15
